# The Role of Ethnicity and Environment in the Regulation of Response to Sensory Stimulus in Children: Protocol and Pilot Findings of a Neurophysiological Study

**DOI:** 10.2196/resprot.8157

**Published:** 2018-01-18

**Authors:** Ivan Neil Gomez, Cynthia YY Lai, Chetwyn CH Chan, Hector WH Tsang

**Affiliations:** ^1^ Department of Rehabilitation Sciences The Hong Kong Polytechnic University Kowloon China (Hong Kong); ^2^ Center for Health Research and Movement Science College of Rehabilitation Sciences University of Santo Tomas Manila Philippines

**Keywords:** ethnicity, environment, children, autonomic nervous system, parasympathetic nervous system, sympathetic nervous system

## Abstract

**Background:**

The ability to regulate the response to sensory stimuli has been associated with successful behavioral patterns necessary for daily activities. However, it is not known whether a child’s ethnicity and environment can influence autonomic regulatory mechanisms.

**Objective:**

This study aims to explore the role of ethnicity and environment in the regulation of responses to sensory stimuli in children.

**Methods:**

In this study, we intend to recruit 128 children from different ethnic groups or environment contexts as follows: (1) 32 typically developing Chinese children living in Hong Kong; (2) 32 typically developing Filipino children living in Hong Kong; (3) 32 typically developing Filipino children who are living in urban areas; and (4) 32 typically developing Filipino children who are living in rural areas in Philippines. Autonomic activity (heart rate variability [HRV] and electrodermal activity [EDA]) will be measured and recorded using Polar H2 heart rate monitor and eSense GSR skin response sensor. Autonomic activity (HRV-low frequency, HRV-high frequency, and EDA) at different conditions between pairwise groupings will be tested using multivariate analysis of variance (MANOVA). All significant levels will be set at *P* ≤.05.

**Results:**

We present the research protocol of this study, as well as a short discussion of the preliminary findings from our pilot data, with consequent power and sample size analysis that informs the appropriate sample needed to test our hypothesis.

**Conclusions:**

This study will increase the understanding on the role of individual differences related to a child’s ethnicity and environment in the regulation of response to sensory stimuli. The findings of this research may further shed light on the evaluation and treatment planning for children across and within cultures.

## Introduction

### Background of the Study

The response to sensory stimuli can be defined as an individual’s reactivity to sensory inputs [1]. Sensory information from the environment is processed and regulated by the individual to support behaviors in everyday situations and development [2,3]. The regulation of response to sensory stimuli entails adjustments of internal measures as a reaction to external demands. Difficulty in the regulation of response to sensory stimuli has been suggested to be prevalent in 40-80% of children with disabilities and in as much as 5-16.5% among typically developing children [4]. The literature that examines the regulation of response to sensory stimuli seems to largely use behavioral and physiological measures [5]. Preliminary evidence suggests that atypical sensory-related behaviors are related to underlying physiologic autonomic regulation [5-9]. Individual differences (ie, age, gender, ethnicity, environment, etc) have been suggested to impact both behavioral and physiological responses [10]. In this research, we examine the role of ethnicity and environment in the regulation of response to sensory stimuli in children using a neurophysiological framework, conceptualized to reflect the information-processing model, which assumes that sensorial information from the environment is subject to underlying physiological processes [11,12].

The theory of allostasis and the allostatic load model is intended to be used in this research to theorize the role of ethnicity and environments in the regulation of response toward sensory stimulus. Foundations of the theories on allostasis were supported by evidence from the studies of McEwen [13,14], Sterling and Eyer [15], and McEwen and Wingfield [16]. In allostasis, alteration of the regulatory parameters (eg, by increasing or decreasing the set point of a homeostatic physiological mechanism) allows a person to adapt to environmental challenges and can create a new baseline to maximize the individual’s responses [17,18]. Individual differences shape how individuals respond to external challenges [10,13]. Such individual differences have been categorized to involve expressive-dependent information appraisal and biological embedding [10,13,19]. Expressive-dependent information appraisal suggests that one’s life history, which includes environmental, cultural, social, or economic backgrounds, shaped our abilities to make sense of situations and instilled in us a set of codes that determine how we react or appraise a situation. In this research, expressive-dependent information is conceptualized to be the influences related to one’s environment, due to the geographic niche or the physical landscapes to which a child has been exposed to. Biological embedding is related to the physiological expression of genetic traits exemplified by our body’s condition [10,13]. These individual differences can account for variations in how individuals successfully adapt to the incessant stimulation or challenges in the environment. In this research, these biologically embedded traits are conceptualized to be the influence brought about by a child’s ethnic origins.

The mechanisms underlying the allostatic response draw from physiological perspectives of how the body responds to and regulates itself from external challenges [10,13,17]. In response to environmental challenges (ie, stress, sensory information), mediators of allostasis effected by multiphysiological systems (ie, hormones, parasympathetic nervous system [PNS], sympathetic nervous system [SNS]) are activated in different modes of control (ie, increase, decrease, no change) to meet the needed demands [20]. Although allostasis is responsible for short-term adaptation, survival, and homeostasis, according to McEwen and Wingfield [16], it can potentially produce permanent changes in a person after prolonged exposures termed as allostatic load.

Ethnicity refers to the biomedical origins and genetic similarities among individuals [21-23]. Ethnicity represents biologically embedded genetic traits and its expressions that have been suggested to influence behavioral and physiological responses related to adaptation [19]. A child’s response to sensory information within his environment seems universal and stable across ethnicities. Royeen and Mu [24] implicated no significant differences in the sensory responses between North American and multiethnic European children situated in the United States and Germany, respectively. Conversely, other researchers counter-argued and found influences of ethnicity on children’s regulation of sensory stimuli using behavioral measures [2]. Caron et al [25] obtained some differences on the responses to sensory information between North American and Israeli children in their responses to sensory experiences. Although Tirosh et al [26] suggested that ethnicity can account for significant differences in the regulation of sensory response between 2 different ethnic groups of children living in the same environment, older children from different ethnic-cultural groups exhibit differences in regulatory responses. The evidence on the influence of ethnicity on the regulation of response to sensory stimuli appears to be inconsistent and inconclusive. Furthermore, it is unclear whether such differences are influenced by inherent ethnicity or the environment in context. In this research, we look at the regulation of response to sensory stimuli among a group of children from different ethnicities, while controlling for environment variables. Specifically, to represent such ethnic differences, we recruit Hong Kong-Chinese and Filipino school-aged children living in Hong Kong and the Philippines, respectively.

The environment is the geographic range where a group of individuals exists [27]. Expressive-dependent information appraisal suggests that the responses of an individual are shaped by similar sociocultural traits and experiences shared by a category of people that set apart one group of people from another, who are embedded within similar geographic environments [10,13,19]. Similar sociocultural traits and experiences embedded within comparable geographic environments result in behavioral responses that are shared by a category of people that set apart their group from another [28,29]. These factors within the geographic environments have been implicated to account for differences or associations between individuals from that of a different one [30]. Thus, the geographic environment, such as the country of abode, is likely to influence the response to sensory stimuli among children, suggesting that the geographic environment where a child develops may have the ability to override ethnic influences [31]. The experiences encompassing within the child’s environment, which include the sensory events and stimuli, possibly shape their responses consequently. Previous researchers suggested that differences in beliefs, practices, behaviors, culture, parenting practices, physical landscapes, and sociocultural and economic aspects can explain these differences [10,13,19,28-31]. However, the experiences related to the sensory information within a particular geographic environment may be different. Furthermore, there is a dearth of evidence that further supports this notion. In this research, we conceptually define environment on 2 levels, that is, geographic environments and physical environment, and explore their influence on a child’s ability to regulate sensory stimulus.

The physical environment is defined herein as the objective characteristics of the physical context related to habituation and gradients of man-made or natural structures and components [32]. This is exemplified in the dichotomous characteristics of urban and rural settings, further operationalized by Perloff [33]. An urban physical environment describes a developed metropolitan setting characterized by a density of man-made human structures (ie, houses, buildings, bridges, railways). On the other hand, a rural environment may be viewed as an open strip of natural environment (ie, agricultural, coastal, mountainous) inhabited by fewer humans with lesser man-made infrastructures. The physical environment can greatly impact how individuals regulate responses to the external world [34]. In comparing urban and nature environments, it was found that the latter produces less physiological arousal and attentional demands [35,36]. Among Taiwanese children, Lin et al [37] proposed that urban and rural children have different performances in the regulation of responses to sensory stimuli. Tirosh et al [26] similarly found urban-rural differences and specifically suggested sociocultural influences (ie, maternal education, cultural differences) as a moderating factor in the regulation of response to sensory stimuli among rural-dwelling children. On the other hand, a different group of researchers suggested that the physical features of the dwelling spaces of children can facilitate better regulation of response to sensory information among urban-dwelling children as reflected by significantly superior perception, perceptual-motor, and cognitive performance [38]. The physical characteristics of the living environment, which include the noise level, visual stimuli related to infrastructures or nature landscapes, pollution, topography, and space, to name a few, have been previously suggested to contribute to possible differences [10,13,19,34-37]. At this time, it is uncertain whether the variation of responses toward sensory stimulation in people of the same ethnicity across regions is related to their physical environment or other factors.

The findings of previous researchers on the influence of ethnicity and environment appear inconclusive. One caveat of previous studies is that the measurement of responses toward the stimuli was not under a controlled environment. The environmental conditions when one experiences sensory stimulation may be varied between levels of the environment (ie, physical space, sound environment, visual stimuli in the environment). In this research, we classify environments on 2 levels: geographic and physical environments. Geographic environments refer to the geographic range where a group of individuals exists [27]. The physical environment is defined herein as objective characteristics of the children’s physical context related to habituation and gradients of man-made or natural structures and components represented by urban and rural settings in this research [32]. Thus, the findings of previous studies may have a limitation on explaining the influences of ethnicity and environment. Another limitation of previous studies concerns their methodology, which has adopted mainly behavioral measures as measuring instrument. Behavioral outcomes, whether through clinical observations or parent reports, are unable to provide precise information about the regulation of response to sensory stimuli [5]. Previous research has found initial evidence that associates SNS [39] and PNS autonomic functions [40] to the regulation of response to sensory stimuli. Indexing physiological responses can offer a more sensitive and objective measure of the underlying mechanisms of internal state regulation in response to external sensory challenges [41]. A child’s environment is contextualized within the sensory events and stimuli that are embedded in it, which makes it a variable of interest. To examine the influence of ethnicity and environments on the regulation of response to sensory stimuli, objective neurophysiological outcomes are needed. In this research, the authors examine the regulation of PNS and SNS autonomic measures in response to sensory stimuli among a group of children.

Autonomic regulation refers to the underlying physiological mechanisms mediated by the autonomic nervous system (ANS) and its parasympathetic and sympathetic branches that support regulation of internal responses in the face of external demands to maintain homeostasis [42]. Empirical evidence that supports autonomic regulation of response to sensory stimuli stemmed out from a series of research that hypothesizes differences in PNS or SNS functions in response to a laboratory paradigm that presents a series of sensory stimuli among children with and without known problems in the regulation of response toward sensory stimuli. In a systematic review that explores the extant literature in the behavioral and physiological regulation of response to sensory stimuli among children, Gomez et al [43] have suggested methodological inconsistencies. For instance, the prevalent use of a single autonomic physiological measure was commonly observed. The group of Schaaf [40,44,45] has suggested PNS functions, specifically the cardiac vagal tone, whereas Miller’s group [46-48] worked on the SNS functions using electrodermal activity (EDA) as an autonomic measure in their research. Even when the same autonomic measure was used, specific parameters and its operational definition appear to vary across studies. However, the use of a single physiologic measure to represent the complexity of autonomic functions should be approached carefully. Thus, researchers need to refocus the choice of autonomic measure to represent the dynamic and interrelated allostatic relationship between the PNS and SNS [45]. Another observation noted was on the choice of laboratory paradigm across various studies. The Sensory Challenge Protocol (SCP), as initially described by Miller and colleagues [46], is a child-friendly laboratory paradigm suggested to be a reliable measure to quantify a child’s physiologic regulation of response to multisensorial stimuli and has been recommended for its ability to reliably quantify physiologic regulation of response to sensory stimuli [49]. However, the use of a multidomain sensory paradigm may not always be suitable. Our recent literature review urges the use of modality-specific measurements to improve the sensitivity of measures [43]. The auditory modality seems to offer a more sensitive stimulus to elicit physiological responses [6,50]. In any case, studies that refer to SCP must modify with caution and decisions must be rationale driven, supported with evidence from the physiological literature. Additionally, autonomic activity is influenced by external environmental factors related to temperature, humidity, and noise level, among others [51-53]. In this study, we use an auditory paradigm to represent external challenges to elicit a multiphysiological response as indexed by the SNS and PNS allostatic mediators. Furthermore, environmental factors during the experimental procedures are discretely controlled during the laboratory paradigm experiment and explicitly reported.

Measures for ANS could provide an objective measure of internal state and the capacity in regulation. In this study, heart rate variability (HRV) and EDA will be measured. Both measures are recommended to be safe, efficient, and noninvasive and objective measures of autonomic activity. HRV has been considered as a promising marker for autonomic activity [51] and has been an increasing outcome option in providing insight into individual differences in autonomic response [41]. HRV indices of low frequency (LF) and high frequency (HF) bands represent the activity of SNS and PNS, respectively. Because HRV can measure both SNS and PNS simultaneously, this study will use HRV as one of our measures. Nevertheless, it has been argued whether the value of LF is a pure measure of SNS functions [52]. Therefore, other than HRV, EDA will also be a supplementary measurement in this proposed research. EDA is a well-recognized sensitive indicator of SNS activity and has been used in identifying physiologic activities related to sensory stimuli responses [53]. Considering the nature of data processing, the measurement of HRV and EDA is suitable for an experiment using block design as in this proposed study.

Sensorial input from the external environment is regulated through neurophysiological mechanisms where reactivity is determined, conventionally, in the form of adaptive responses. The ANS response is conceptualized in this research to include the child’s physiological capacity to receive sensory information, react to such information, and recover thereafter. The capacity to receive sensory information can be represented by the resting baseline (sometimes called as resting, basal, or baseline) condition of the ANS activity and is indexed to establish basal levels to which changes in experimental conditions can be referred to [54-56]. The doctrine of autonomic constraint, as initially proposed by Berntson et al [20], suggests that the ANS activity capacity of magnitude change depends on the set starting point. This starting point, as represented by the resting autonomic functions of PNS and SNS, may be involved in regulation of responses. Exposure to sensory stimuli entails physiological reactions from ANS, which has been previously suggested to be the first system to respond to external challenges. This is characterized by the physiological reactivity during the stimulation condition where patterns of magnitude changes from resting baseline conditions to the point of stimulus presentations are referred to [54-56]. During stimulation conditions, the ANS function levels are measured, but other measures are likewise suggested, such as the mean difference between values at the stimulation and resting baseline conditions [57] or the magnitude of change [58]. The recovery condition is roughly described as the temporal point at which experimental stimulation ceases and conditions similar to the resting baseline conditions are used. To an extent, this condition can indicate adaptability of the child after stimulation. In this research, the concept of allostasis is used to better explain the adaptation that is manifested during the recovery condition. Allostasis allows adapting one’s self to the changes in their environment [59] through autonomic responses that can be facilitated by mediators of allostasis.

ANS is the first physiological axis to be activated in response to an environmental challenge. Mediators of allostasis can include heart rate, respiratory rate, blood pressure, cardiac output, and EDA [60,61]. The activity of allostasis mediators is integrated and cross-regulated and reflects components of a single functional system even with anatomical and physiological differences [62-64]. Thus, regulatory mechanisms involved in the allostatic process are described as a sequential or synchronized event between different mediators [65,66]. However, previous research seems to index singular mediators (ie, PNS or SNS only) of allostasis and may not reflect the inherent nature of how subsystems are interrelated with each other. We apply such concepts in this research by looking at the multivariate combination of HRV and EDA to represent allostasis, the mechanism related to the regulation of response toward sensory stimulus in children. The allostatic load is used to represent the influence of ethnicity and environments among the groups of children recruited in this study.

Sensory information from the environment is processed and regulated by the individual to support behaviors in everyday situations and development. The concept of allostasis suggests that adaptation to external demands entails internal regulation of physiological parameters. Individual differences shape how individuals respond to external challenges. Such individual differences have been categorized to involve expressive-dependent information appraisal and biological embedding. Ethnicity represents a proxy measure of biologically embedded genetic traits that differentiates one ethnic group from another (ie, Chinese, Filipino). The environment represents 2 levels of experience-dependent information based on (1) geographic environments, which take on the geographic niche of habituation represented by the country of living (ie, Hong Kong and the Philippines), and (2) physical environment, which explains the physical landscape features of the environmental habituation represented by urban and rural settings. In this research, we recruit children from similar ethnic origins (ie, Filipino) living in different geographic environments (ie, Hong Kong and the Philippines) and physical environments (ie, urban and rural Philippines). Neurophysiological measures of HRV and EDA that represent the interrelated activities of PNS and SNS will be primarily measured, alongside usual behavioral measures of how children adaptively regulate responses to sensory stimuli.

### Research Question and Motivation

The motivation for this research stems from the dearth of evidence in the literature that supports the role of individual differences in the regulation of response to sensory stimuli in children that use neurophysiological outcomes. Our research question, therefore: *do ethnicity and environments influence the regulation of response to sensory stimuli in children?* The findings in this research can have implications in cross-culturally appropriate evaluation measures and intervention procedures that are relevant to the regulation of responses to sensory stimuli.

### Research Hypothesis

This research study is an exploratory inquiry on the role of ethnicity and environments in the regulation of response to sensory stimuli among children by using neurophysiological outcome measures. Supported by behavioral studies and the concept of allostasis as applied in this research, the researchers hypothesize that there is significant difference in the regulation of response to sensory stimuli in (1) children from different ethnicities living within the same geographic and physical environments, (2) children from similar ethnicities and physical environments living in different geographic environments, (3) children from different ethnicities and geographic environments living in similar physical environments, and (4) children from similar ethnicities and geographic environments living in different physical environments.

### Research Aims

Considering the available evidence in the current literature and our perceived gaps in the knowledge related to our topic, in this study, we study the role of ethnicity and environment in the regulation of response to sensory stimuli among children from a neurophysiological perspective by applying the theory of allostasis. We aim to explore differences in the regulation of response to sensory stimuli in children through their ethnicity (as proxy measure of genetic influence) and environments (as a general measure of beliefs, practices, behaviors, culture, parenting practices, physical landscapes, and sociocultural and economic aspects). Specific to this manuscript, the authors also aim to determine the ample sample size needed for the main study, based on the pilot data presented.

## Methods

### Ethical Considerations

Ethical approval was obtained from the Hong Kong Polytechnic University, Human Subjects Ethics Sub-committee (Hong Kong, SAR), with reference number HSEARS20150316001, and from the University of Santo Tomas-College of Rehabilitation Science (Philippines) with protocol no: FI-2015-02. Before the data-gathering procedure, assent forms and parental consent forms will be distributed to the participants of the study and their respective parents. Whenever the discomfort could not be tolerated by the child, the testing procedure was stopped.

### Participants

Using a cross-sectional study design, this study compares the autonomic activity in different experimental conditions (resting baseline condition, stimulation condition, and recovery condition) among 4 groups of participants, comprising 2 groups of typically developing children, (1) from different ethnicities (Chinese children in Hong Kong, Filipino children in the Philippines-Urban area); (2) from different geographic environments (Filipino children in Hong Kong, Filipino children in the Philippines-Urban area); (3) from different ethnicity and geographic environments (Chinese children in Hong Kong and Filipino children in the Philippines-Urban area); and (4) from different physical environments (Philippines-Urban area, Philippines-Rural area). Participants will be typically developing boys and girls, aged 7-12 years with no known developmental, neurological, or medical condition. The sample size of n=32 for each group was initially estimated based on the mean change of ANS indices of a previous study with similar but not the same experiment [67] by using the effect size of 0.8 for group differences with the alpha level at .05.

### Assessments and Measures

#### Behavioral Measures

To measure a child’s behavioral patterns in response to sensory stimulus in his daily activities, the original Sensory Profile [2] and Chinese Sensory Profile [68] are used. The original Sensory Profile, which was developed and validated by Dunn [2], is a 125-item questionnaire that is completed by the caregiver. The Chinese Sensory Profile is a 100-item parent-report measure used among children and uses the same rating scale as the original one. We extracted the similar items from these 2 versions; the computed total and subscale scores will be subjected to intraclass correlation coefficient computation later on. The Sensory Profile has been used in both normative and clinical population in screening for sensory modulation disorders. Higher scores indicate lesser sensory behavior issues, whereas lower scores, typically seen in clinical populations, suggest the prevalence of sensory issues. Because the recruited participants in this study are assumed to be typically developing, statistical analysis may be confounded. We will further use other behavioral measures that can represent behavioral responses. Temperament and resilience were chosen, as this gives unbiased composite scores from which we can ascertain whether sensory behavior responses are deemed to be issues or just a trait pattern.

To measure temperament, the Temperament in Middle Childhood Questionnaire [69] and Early Adolescent Temperament Questionnaire [70] will be used. Both are psychometrically sound measures that are answered by parents to describe their children’s traits [70,71]. We will use the translated and validated Chinese version (personal communication by Samuel Putnam, 2015) for the relevant populations. Three factors will be used in the group analysis: negative effect, surgency, and effortful control.

To measure resilience, the Child and Youth Resilience Measure (CYRM) [72], which was established through a process of interviews with youth and adults in countries around the world, was completed by the participants’ parents. Previous research has suggested that ethnicity and environmental experiences influence the development of resilient behaviors [73,74] and that it is likely moderated by autonomic activity [75-77]. We included resilience as a behavioral outcome measure to determine whether group differences in autonomic activity across conditions can be explained by our main dependent variables (DVs, ie, ethnicity, environment), or other factors, for which we may try to control. This research uses the 28-item (>10 years of age) or the 26-item (5-9 years of age) Person-Most-Knowledgeable questionnaire version, which was developed and validated by Ungar and Liebenberg [78]. The similar items between the 2 versions will be used for analysis. Although CYRM is originally in English, a translated Chinese version was recommended and provided by the authors and was used among Chinese participants in this research (personal communication, Linda Liebenberg, 2015).

#### Neurophysiological Measures

HRV enables this research to look at the activities of PNS, SNS, and their interactions across different experimental conditions (ie, resting baseline, stimulation, recovery conditions). For this study, Polar H2 Heart Rate Monitor (Polar, Finland) is used to measure HRV at 5 kHz sampling rate. There is some evidence that supports the use of Polar heart rate monitors as a valid instrument to measure HRV [79], and ultimately follow the set guidelines for HRV measurement and research [51].

EDA is an autonomic measure of SNS. Because LF bands of HRV have been contested as a true measure of SNS activity, EDA is used as an adjunct measure in this research. The eSense Skin Response-GSR sensor (Mindfield, Germany) measures skin conductance level (SCL) and response (SCR) with a sampling rate of 5 Hz and a resolution of 18 bit, using direct current. The device performs exosomatic measurement with a direct current of 0.50 V through two 5-mm silver-silver chloride (Ag/AgCl) electrode. Although fairly new, the specifications of eSense meet the general publication recommendations for EDA research [52,53].

### Procedures and Paradigm

**Figure 1:**
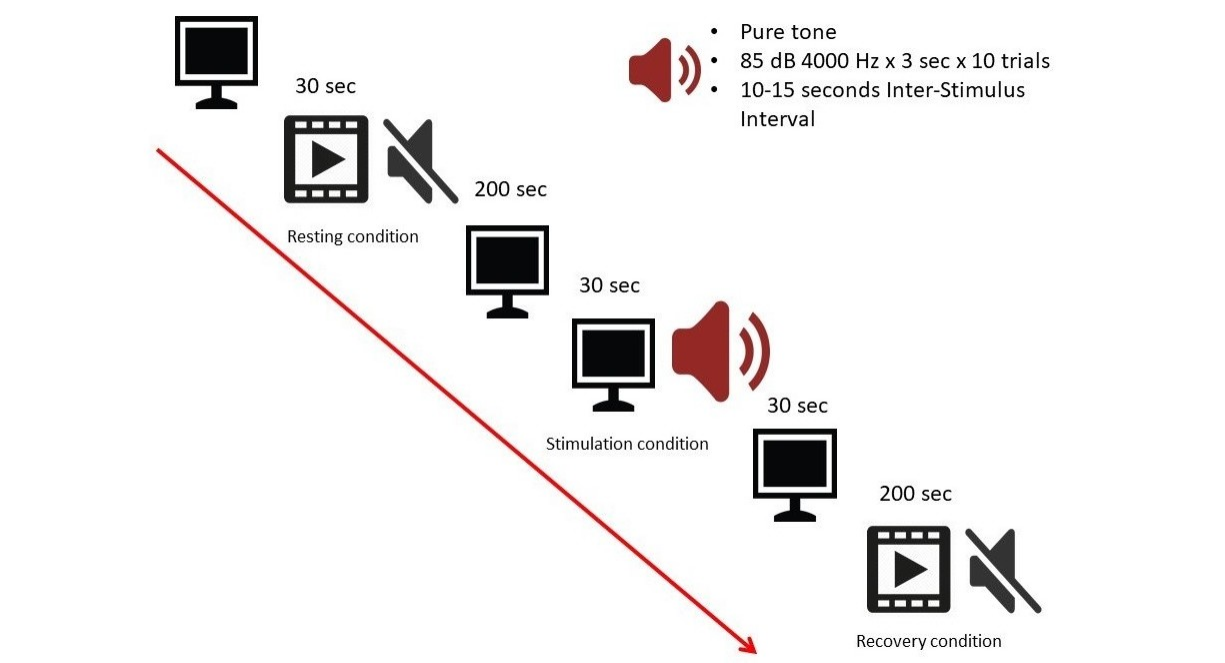
Laboratory paradigm.

The experimental protocol used in this part of the study is adapted from SCP, first employed by Miller et al [46], with a few modifications based on a systematic review by Gomez et al [43]. Figure 1 presents the experimental paradigm used in this study. Instead of using various multisensory stimuli, we opt for an auditory stimulus to represent external challenges to the allostatic response. Furthermore, we use 3 conditions, instead of the original resting baseline and stimulation conditions. A 3-min resting baseline period is used to allow sufficient data time points necessary for analysis of autonomic responses (HRV-HF [normalized units (n.u.)] and LF (n.u.); EDA-SCL) [51-53]. During this time, a silent cartoon movie is shown to the child for 200 seconds. At the condition of sensory stimulation, the screen turns blank and the participant receives a block of passive auditory sensory stimulation (a block of 10 trials of a 4 KHz pure tone at 85 dB; each trial lasting for 3 seconds) with a pseudorandomized interstimulus interval of 10 to 15 seconds to avoid adaptation to stimuli. The auditory stimulation condition lasts for approximately 160 seconds. The use of a pure tone is another modification of this research, used from the original fire engine sound, to reduce any emotional responses to familiarity with the stimuli. The loudness is reduced from the original 95 dB to 85 dB to ensure that the intended physiological response is recorded and no other internal events related to louder auditory stimuli [80]. At the recovery condition, the same silent cartoon movie is played for 200 seconds. Previously, the recovery condition was not a part of the original SCP; however, it has been added and adapted in more recent literature. Such recovery conditions may represent physiological regulation related to adaptation, further described in the concept of allostasis. This entire procedure takes around 12 min. The experimental protocol, including the generation of the pure tone for auditory stimuli, is developed and executed using LabView (National Instrument, USA).

ANS measures are strongly affected by environmental factors. Thus, in all studies, regardless of the country (Hong Kong or Philippines) where the research is conducted, the same procedures are implemented. Similarly, other factors that influence physiological responses can likewise influence the ANS response. To control for this, parents of the participants are instructed with experiment preparatory reminders: no intake of caffeinated drinks 4 hours before the scheduled testing; no intake of food 1.5 hours before the scheduled testing; no rigorous physical exercise before the scheduled testing; and no treatments/interventions (ie, sensory integration therapy, craniosacral therapy, acupuncture, or any other procedures that could influence ANS activity) 24 hours before the scheduled testing.

During the experimental testing, the body mass index (BMI) is ascertained through measurement of the participants’ height and weight. The Polar H2 chest strap is securely fastened just below the chest muscles. The finger cuffs are fixed around the medial phalanx of the index and middle fingers, opposite the laterality of the participant’s hand. The child sits on a comfortable child-sized chair, facing a 19-inch monitor 3 feet away from him. A set of over-ear open headphones is placed over the child’s ears. During the actual experimental conditions, the room is dimmed to 10 lux (illumination level), temperature set to 23 to 25°C, humidity level at 60 to 80%, and background noise level at 40 to 45 dB. The researcher is located 60 to 80 cm away to the left of the participant, keeping interaction to a minimal. Testing procedures in this research are adapted from a similar previous study by Lai [63] and a systematic review by Gomez et al [43]. These conditions are kept constant across all testings.

### Data Analysis

#### Neurophysiological Data

HRV is acquired using Polar H2 heart rate monitor, recorded in real time, and simultaneously stored using the Polar Trainer 5, directed through infrared signals. The software for HRV analysis used was aHRV (Nevrokard, Slovenia), which uses the current guidelines for HRV analysis [47]. Raw data were converted into aHRV tachogram files. Using the researcher’s observation notes, tachograms are subjected to visual analysis, identifying ectopic beats, movement artifacts, and abnormal noise signals. HRV files are then epoched into specific time events in the experimental paradigm. The epoched tachograms are subjected to correction of artifacts following the guidelines used by Task Force of the European Society of Cardiology [51]. In identifying artifacts for the short-term recordings, aHRV compared values to 20% under or over the mean of the preceding 25 beats [51]. Identified noise artifacts are then edited using proper interpolation, keeping as much of the integrity of the data sample. Data with more than 3% correction from the total normalized HRV data samples were discarded from the analysis. A 512-point Fast Fourier Transform was generated using a Hanning window to minimize spectral leakage at truncated data segments. Frequency domain analysis covered low (LF: 0.04-0.15 Hz) and high (HF:0.15-0.40 Hz) frequency components in its normalized units. The normalized units of LF were used as representatives of sympathetic modulation activity (predominantly), whereas the normalized units of HF were used as representatives of parasympathetic modulation activity using the following formula: LF *or* HF/(total power−VLF)×100, where VLF stands for very low-frequency band.

Ledalab version v.3.2.9, a MATLAB-based computer program, is used to extract EDA using a continuous decomposition analysis (CDA) method [81]. Epoched EDA data are then preprocessed individually using visual analysis and subsequent data grooming to reduce noise, which includes manual smoothing using a 5-second Hann window [82,83] and a filter of a unidirectional first-order Butterworth low-pass filter with a similar cutoff frequency of 5 Hz [83,84]. The traced EDA artifacts are then corrected using a spline interpolation within a 5-second pre/post parameter [85]. The CDA method uses parameters based on the previous works of Benedek and Kaernbach [81], which optimizes the EDA data and applies a 0.2-Hz Gaussian smoothing window within a 10-second grid size to detect significant peaks of >0.05 µS [86]. For the resting baseline and recovery conditions, SCL is extracted by using the *CDA.Tonic* parameter, which computes for the mean tonic EDA within the epoched response window (150-second block), in an aggregated 10-second moving within-window averaging method. In the case of processing the EDA stimulation condition data, a similar CDA method is used (Bateman functions comprising onset, amplitude, 1 and 2 parameters). The software can identify significant peaks of >0.05 µS [87], within a response window of 1 to 4 seconds poststimulus [82,88] across the stimulation condition block that consisted of 10 trials. To represent the sympathetic activity in the form of EDA, *CDA.SCR* was identified [82,87], which was then averaged across trials (0 responses not included) within the stimulation block condition as estimate of mean sympathetic activity, reflecting the individual’s amplitude of responsiveness to sensory stimulus. To remove the impact of within and between-subject variance, analysis was performed using z-transformed individual *CDA.SCR* scores [82]. Furthermore, to correct for skewed distribution of skin conductance data scores and to meet assumptions required for parametric statistical analyses, normalization of SCL and SCR data for each intersection was performed using square root transformation [83,89-91]. This logarithmic transformation is determined as follows: *√(CDA.Tonic)* and *√(CDA.SCR+1)*.

#### Statistical Data

Descriptive statistics involving measures of central tendencies, variation, and dispersion are used to describe the salient characteristics of the data gathered. Demographic data related to participant gender, age, BMI, type of school (ie, public, private), number of parents working, primary caregiver, highest educational attainment of caregiver, family income, and parent’s occupation will be gathered and considered in further statistical analyses. The Shapiro-Wilk test of normal is used to determine normality at baseline of the characteristics of the participants included in the study. Univariate and multivariate tests are likewise used to determine whether there are group and subgroup baseline similarities at critical alpha=.05. Variables deemed as non-normally distributed or significantly different at baseline are used as covariates. General linear modeling is used to determine whether effects of ethnicity or environment (used as the independent variables [IV] in this study) can explain differences in the behavioral measures of the participants at a critical value of alpha=.05. Autonomic regulation is represented in this research as HF (n.u.) and LF (n.u.) of HRV and mean EDA (SCL/SCR) values as multivariates. Multivariate analysis of variance (MANOVA) tests will be used to determine whether effects of ethnicity or environment (used as IV in this study) can explain differences in the multivariate set of DVs, across the different research aims, with the different pairwise combinations in between 3 events (resting baseline, stimulation, and recovery). Effect size is interpreted using the values of Cohen *d*. Statistical analysis will be computed using SPSS version 23.0.

Specific to this report, sample size and power analysis are performed using G*Power version 3.1.9.2 [91,92]. Post hoc analysis of computed achieved power of the pilot data will be ascertained to determine the needed effect size for a subsequent a priori computation of required sample size for MANOVA tests.

## Results

For this report, we present data from our pilot data which include 30 participants (n=30) of 2 groups of children with controlled ethnicity (Filipino) but living in different physical environments (urban and rural settings) during the resting baseline phase of our experimental paradigm. The pilot samples are controlled for gender and age-matched. Table 1 presents a summary of the demographics and statistics of the variables included in the analysis for this paper.

### Group Differences: MANOVA

Preliminary data from 30 gender-controlled and age-matched participants with a mean age of 8.667 (SD 1.759) are analyzed for the influence of physical environments on the neurophysiological regulation of response to sensory stimuli using the HF (n.u.) and LF (n.u.) bands of HRV and EDA-SCL during the resting baseline condition as DVs. On the basis of the Shapiro-Wilk test and independent samples *t* test, BMI was normally distributed (*P*=.149) and similar at baseline (*P*=.57) between urban- and rural-dwelling participants.

The mean HF (n.u.) for the resting baseline condition was computed at 62.301 (SD 15.903) for urban-dwelling children, which is higher than the rural-dwelling group (mean 52.096, SD 15.125). This is the other way around for the LF (n.u.), where the rural-dwelling group (mean 29.228, SD 10.639) fared better than the urban-dwelling group (mean 24.171, SD 9.370). EDA-SCL showed almost similar values, with the urban group having slightly higher values (mean 1.929, SD 0.600) compared with their rural group (mean 1.900, SD 0.363).

**Table 1:** Summary of demographics and statistical results.

Demographics		Urban (N=15)	Rural (N=15)	Total (N=30)	Shapiro-Wilk test	*t* test	*F* test			
							Pillai *V*	*F*	*P*	*d*
**Type of school, n (%)**										
	Public school	12 (80)	N/A^b^	N/A						
	Private school	N/A	15 (100)	17 (57)						
**Number of parents working, n (%)**										
	Both parents	10 (67)	13 (87)	23 (80)						
**Primary caregiver, n (%)**										
	Mother	13 (87)	12 (80)	25 (83)						
**Primary caregiver’s highest educational level, n (%)**			College degree	College degree						
	College degree	8 (53)	10 (67)	18 (60)						
Age in years, mean (SD)		8.667 (1.759)	8.667 (1.759)	8.667 (1.759)		1.000				
BMI^c^, mean (SD)		16.145 (3.722)	15.467 (2.683)	15.806 (3.207)	0.149	0.572				
HF (n.u.)^d^, mean (SD)		62.301 (15.903)	52.096 (15.125)	57.199 (16.108)	0.504		0.107	1.033	.394	0.692
LF (n.u.)^e^, mean (SD)		24.171 (9.370)	29.228 (10.639)	26.700 (10.181)	0.448					
SCL^f^ (µS), mean (SD)		1.929 (0.600)	1.900 (0.363)	1.914 (0.488)	0.397					

^a^N/A: not applicable.

^b^BMI: body mass index.

^c^HF (n.u.): high frequency (normalized unit).

^d^LF (n.u.): low frequency (normalized unit).

^e^SCL: EDA-Skin Conductance Level.

**Table 2:** Sample size analysis protocol.

Analysis^a^		Results
**Input**		
	Effect size *f*^2^(*V*)	0.0625
	α err prob^b^	0.0500
	Power (1- *β* err prob)^c^	0.8000
	Number of groups	4
	Response variables	3
**Output**		
	Noncentrality parameter *λ*	16.5000
	Critical *F*	1.9171
	Numerator df	9.0000
	Denominator df	252.0000
	Total sample size	88
	Actual power	0.8094
	Pillai *V*	0.1765

^a^Computation was based on *F* tests–MANOVA: Global effects with analysis of a priori to compute for the required sample size.

^b^This is the critical value.

^c^This is the specific formula to compute for the power.

Using physical environment (urban or rural setting) as IV and the values at resting conditions of the LF (n.u.), HF (n.u.), and SCL as DV, a multivariate analysis of covariance (MANCOVA) test, specifically Pillai *V*, was performed using BMI as a covariate. In the series of analyses of variance (ANOVAs) performed, results of the multivariate tests confirm that the differences in the measures of autonomic regulation considered as DV among the 2 sampled ethnic groups in this study are not significant using a critical alpha of .05 (*V*=0.107, *F*_3,26_=1.033, *P*=.39, *d*=0.692). A univariate analysis of individual DVs reveals similar nonsignificant differences between LF (n.u.) (*F*_1,30_=1.909, *P*=.18, *d*=.523), HF (n.u.) (*F*_1,30_=3.243, *P*=.08, *d*=0.681), and SCL (*F*_1,30_=0.025, *P*=.87, *d*=0.063) of the sampled groups. It remains unclear whether the sample size in our pilot findings confounded the influence of the physical environment of the participants in explaining differences in the autonomic regulation of response to sensory stimuli.. Thus, a subsequent power analysis of the sample reported is performed.

### Power and Sample Size Analysis

A post hoc analysis using the results of the MANOVA test for the reported sample population on our preliminary data was conducted using G*Power version 3.1.9.2. Statistical power was computed using a sample of n=30 and the identified 3 DVs at resting baseline condition. Alpha level was set at alpha=<.05. The results of our post hoc analyses suggest that our effect size (*f*^2^=0.120) is considerably in the medium range [93], whereas power was low at 0.283. This is considerably different from the initial set sample size of n=32 (alpha=.05, *d*=0.80, *P*=.80). With this in mind, we then recomputed the required sample size using a priori methods, setting a moderately large effect size of *f*^2^=0.625 and a power of 0.80 for the needed 4 groups among 3 response variables to be used as DVs. Our post hoc analysis suggests that the current samples (n=15 per group) included in our pilot findings are underpowered; hence, significant effects may not yet be seen. On the basis of our results, the needed sample for this research is computed at n=22 per group (Table 2).

## Discussion

Our initial data suggest that ample sample size is needed to demonstrate our concepts and prove our hypothesis, given our methods. Although our initial findings currently yield nonsignificant results, the lack of differences in the regulation of response between our sampled groups might be due to underpowered sample size; hence, the researchers shall be guided with the recommended ample sample size in the offing.

Although there is much that remains to be done, our work has future important findings in the field. The development of the ability to regulate responses to sensory stimuli does not occur in a vacuum, and some other variables (ie, cultural, anthropological, socioeconomic) may likely account for variations. Although we will collect several measures of socioeconomic status (ie, school attended, whether public or private; number of parents working; primary caregiver; highest educational attainment of caregiver; family income; and parent’s occupation) and proxy cultural measures (ie, parent-reported behavioral profiles of the child) and try our best to control for their confounding effects through sample stratification and statistical measures, their main effects are beyond the scope of this research. In this research, we mainly aimed to explore the influence of ethnicity and environment to explain differences in the regulation of response to sensory stimuli among typically developing school-aged children through indexing physiological autonomic measures from a biomedical and neurophysiological perspective.

Regulation of sensory responses from the environment is essential in daily activities. Some children may have difficulty in processing sensory information. However, the underlying mechanism contributed to the regulation of behavior toward sensory stimulation is not clearly understood yet. The answer that our research will eventually provide has several implications. First, our findings may further strengthen the role of individual differences on behavioral and physiological responses, adding to the body of literature that supports the theory of allostasis. Second, our future findings may influence the reconceptualization of the neurophysiological mechanisms behind the regulation of response to sensory stimuli. Third, if our hypotheses are correct, then our findings may influence the review and further development of behavioral outcome measures of regulation of response to sensory stimuli to constitute questionnaire constructs related to physiological symptoms, rather than on purely behavioral ones alone. Finally, this research may further inform clinical practice on the importance that a child’s external environment may play in the regulation of response to sensory stimuli, and consequently, consider these factors in intervention planning for children from clinical populations with diverse ethnic environments.

## Abbreviations

Ag/AgCl: silver-silver chloride
ANOVAs: analyses of variance
ANS: autonomic nervous system
BMI: body mass index
CDA: continuous decomposition analysis
CYRM: child and youth resilience measure
DV: dependent variable
EDA: electrodermal activity
HF: high frequency
HF (n.u.): high frequency (normalized unit)
HRV: heart rate variability
IV: independent variable
LF: low frequency
LF (n.u.): low frequency (normalized unit)
MANCOVA: multivariate analysis of covariance
MANOVA: multivariate analysis of variance
PNS: parasympathetic nervous system
SCL: skin conductance level
SCP: Sensory Challenge Protocol
SCR: skin conductance response
SNS: sympathetic nervous system
